# Influence of Cognitive Reserve on Cognitive Trajectories: Role of Brain Pathologies

**DOI:** 10.1212/WNL.0000000000012728

**Published:** 2021-10-26

**Authors:** Xuerui Li, Ruixue Song, Xiuying Qi, Hui Xu, Wenzhe Yang, Miia Kivipelto, David A. Bennett, Weili Xu

**Affiliations:** From the Department of Epidemiology and Biostatistics (X.L., R.S., X.Q., W.Y., W.X.), School of Public Health, Tianjin Medical University; Tianjin Key Laboratory of Environment, Nutrition and Public Health (X.L., R.S., X.Q., W.Y., W.X.); Center for International Collaborative Research on Environment, Nutrition and Public Health (X.L., R.S., X.Q., W.Y., W.X.), Tianjin; Shandong Provincial Clinical Research Center for Emergency and Critical Care Medicine (R.S.), Institute of Emergency and Critical Care Medicine of Shandong University, Qilu Hospital of Shandong University, Jinan; Big Data and Engineering Research Center (H.X.), Beijing Children's Hospital, Capital Medical University, National Center for Children's Health, China; Division of Clinical Geriatrics, Center for Alzheimer Research (M.K.), and Aging Research Center (W.X.), Department of Neurobiology, Care Sciences and Society, Karolinska Institutet; Theme Aging (M.K.), Karolinska University Hospital, Stockholm, Sweden; Ageing and Epidemiology (AGE) Research Unit (M.K.), School of Public Health, Imperial College London, UK; and Rush Alzheimer's Disease Center (D.A.B.), Rush University Medical Center, Chicago, IL.

## Abstract

**Background and Objectives:**

Evidence on the association of cognitive reserve (CR) with the cognitive trajectories is limited. We aimed to examine the influence of CR indicator on domain-specific cognitive trajectories taking brain pathologies into account.

**Methods:**

Within the Rush Memory and Aging Project, 1,697 participants without dementia (mean age 79.6 years) were followed up to 21 years. CR indicator encompassing education, early-life, mid-life, and late-life cognitive activities and late-life social activity was ascertained at baseline and categorized as tertiles (lowest, middle, and highest). Global cognition, episodic memory, semantic memory, working memory, visuospatial ability, and perceptual speed were assessed annually with 19 tests, from which composite scores were derived. During the follow-up, 648 participants died and underwent autopsies to evaluate brain pathologies. Data were analyzed using linear mixed-effect models.

**Results:**

Among the participants, the score of the CR indicator ranged from −8.00 to 5.74 (mean 0.00 ± 2.23). In multi-adjusted mixed-effect models, compared to the lowest CR, the highest was related to a slower decline in global cognition (β = 0.028, 95% confidence interval [CI] 0.012–0.043), episodic memory (β = 0.028, 95% CI 0.010–0.047), and working memory (β = 0.019, 95% CI 0.005–0.033) during the follow-up. In brain pathologic data analysis, the association of the highest CR with cognitive function changes remained significant among participants with high Alzheimer disease pathology or gross infarcts.

**Discussion:**

High CR indicator is associated with preserved global cognitive function, episodic memory, and working memory, even in the presence of brain pathologies. Our findings highlight the important role of high CR accumulation in the prevention of cognitive decline.

With population aging, cognitive impairment and dementia are common among older adults. Although there is a lack of efficacious treatment for dementia, a growing body of evidence suggests that dementia might be prevented by modifying certain risk factors.^1^ Many people may tolerate considerable dementia-related brain pathology without expressing the clinical syndrome of dementia.^2^ The concept of cognitive reserve (CR) explains such tolerance, and various life exposures seem to be associated with resilience against age- or pathology-related impairment of cognitive function.^3^

Educational attainment, socioeconomic status, social engagement, and cognitive activities are often regarded as proxies or measures of CR.^4,5^ Numerous studies have indicated the association between individual CR-related factors and cognitive function in different domains, but with inconsistent findings. Several studies have shown that participants with higher education have better global cognitive function,^6-9^ but not all the domain-specific cognitive functions.^8,10,11^ Two longitudinal studies have suggested that high education may attenuate global cognitive decline in aging,^12,13^ while others showed no association between education and decline in any domain-specific cognitive function.^14,15^ Similarly, cognitive and social activities have been associated with cognitive function in some domains (including perceptual speed, executive function, and episodic memory).^9,16,17^ However, the relationship between cognitive/social stimulating activities and cognitive functioning remains controversial.^18-21^

Emerging evidence has shown that CR is a dynamic construct and influenced by different exposures across the lifespan. Thus, one reserve-enhancing factor at a certain period alone could not fully explain the accumulation of cognitive activities over the life course.^22,23^ Although few studies have suggested an association of high CR accumulated during life course with a decreased dementia risk,^5,24,25^ it is unclear about the association of CR with domain-specific cognitive decline (i.e., early-stage cognitive phenotype). High CR might directly counteract the accumulation of neuropathologies (such as β-amyloid),^26^ or bypass brain pathologies through other pathways to compensate for or cope with brain pathologies to delay dementia onset.^27,28^ Therefore, the role of brain pathologies in the CR-cognitive function association is in debate.

In the present study, we aimed to examine the associations of CR indicator with global and domain-specific cognitive function changes over time and to explore whether brain pathologies may play a role in such associations among dementia-free older adults.

## Methods

### Study Population

**Figure 1:**
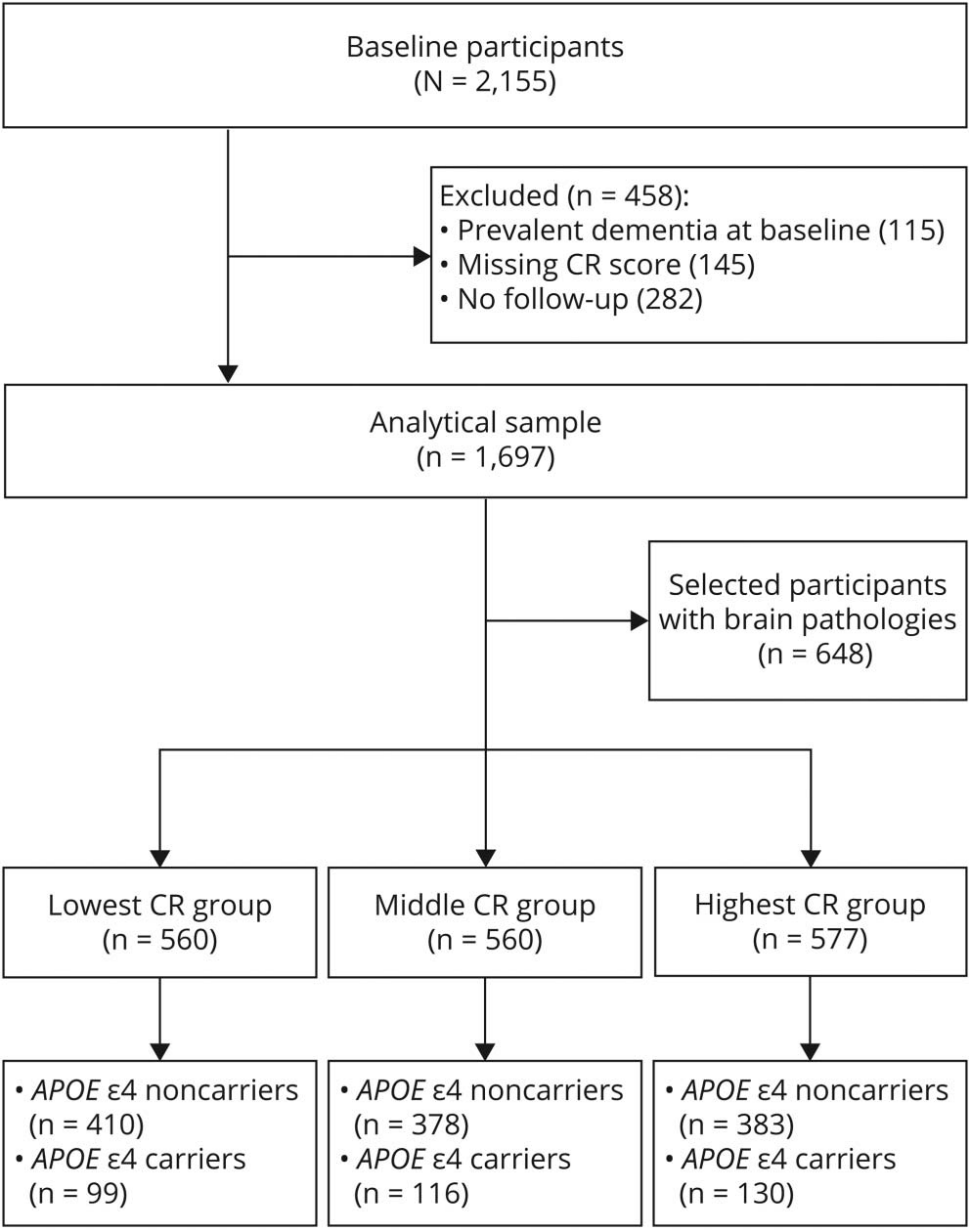
Flowchart of the Study Population CR = cognitive reserve.

The Rush Memory and Aging Project is an ongoing prospective cohort study on aging and dementia.^29^ From 1997 through 2019, a total of 2,155 participants were annually followed up for a maximum of 21 years. Among all participants, we excluded 458 participants, including 115 with prevalent dementia (due to impaired cognitive function, eTable 1, doi.org/10.5061/dryad.59zw3r273), 282 with missing data on CR-related factors, and 145 with missing data on cognitive function during the follow-up. Finally, 1,697 participants remained for the current study. During the follow-up, 795 participants died, of whom 648 (81.5%) underwent autopsies (Figure 1).

### Standard Protocol Approvals, Registrations, and Patient Consents

All participants provided written informed consent before their participation. The study was approved by the Institutional Review Board of Rush University Medical Center and was performed following the ethical standards laid out in the 1964 Declaration of Helsinki and its later amendments. Anatomic Gift Act documentation was obtained from all participants who underwent autopsy. Participants signed a repository consent to allow their data to be shared.

### Data Collection

At the time of enrollment and thereafter, all participants received a comprehensive clinical assessment (including medical and neurologic examinations and comprehensive cognitive function testing) by trained staff. Data on demographic characteristics (e.g., weight and height) and lifestyle factors (including alcohol drinking, smoking, and physical activity) were collected at study entry.

Alcohol drinking was assessed by the average amount (in grams) consumed per day during the past year. Smoking status was grouped into never, former, and current smoking. Physical activity was measured by summing up the hours that the participant engaged in activities per week using questions adapted from the National Health Interview Survey.^30^ Weight and height were measured at study entry by trained staff. Body mass index (BMI) was calculated as weight in kilograms divided by height in meters squared.

Hypertension was ascertained based on systolic blood pressure ≥140 mm Hg or diastolic blood pressure ≥90 mm Hg measured by mercury sphygmomanometer, self-reported history of hypertension, or the use of antihypertension drugs. Diabetes was defined by any of the following situations: hemoglobin A1c ≥6.5%, fasting plasma glucose ≥126 mg/dL, random blood glucose ≥200 mg/dL, history of diabetes, and the use of diabetes medication. Heart diseases (including congestive heart failure, heart attack or coronary, coronary thrombosis, coronary occlusion, and myocardial infarction) were ascertained by self-reported information on medical conditions. Ascertainment of stroke was based on the clinicians' review of self-reported history of stroke or neurologic examination. *APOE* genotype was assessed by Polymorphic DNA Technologies and was dichotomized into ε4 carrier and non-ε4 carrier. Additional details about the data collection can be found online at the Rush Alzheimer's Disease Center Resource Sharing Hub (radc.rush.edu/docs/var/variables.htm).

### Assessment of CR Indicators

Information on CR-related factors (including education, early-life, mid-life, and late-life cognitive activities, late-life social activity, and late-life social network) was collected through personal interviews at study entry. The structured questionnaires on these factors were described in detail previously.^5,31^

Years of education were acquired based on the number of years of regular school reported at baseline.^32^ Cognitive activities in early life (including childhood [aged 6–12 years] and young adulthood [aged approximately 18 years]), midlife (aged approximately 40 years), and late life (during the past year of study enrollment) were assessed at baseline using a 37-item questionnaire of the frequency of participation in cognitively stimulating activities in the corresponding periods. Item scores in corresponding periods were averaged to yield cognitive activity measures (the score of early-life cognitive activity was obtained by further averaging the scores of cognitive activities in childhood and young adulthood).^33^ Higher scores indicate more participation in cognitive activities.

Late-life social activity was assessed with a 6-item scale questionnaire describing how often participants engaged in common types of activities that involved social interaction during the past year. The item scores were averaged to generate the composite measure of social activity and higher scores indicated more involvement in social activity.^34,35^ Late-life social network was quantified by standard questions regarding the number of children, family, and friends that each participant had and how often they interacted with them.^36^

The CR indicator was constructed using structural equation modeling (SEM).^31^ In the initial analysis of latent variables, education, early-/mid-/late-life cognitive activities, and late-life social activity and social network were included in the structural equation. However, the factor loading of social network was small (0.191) and the fitting degree of the equation was unsatisfactory, therefore late-life social network was excluded from the equation. Also, we intended to include physical activity in the SEM when initially creating CR indicator, but its factor loading was low (0.105), leading to an unsatisfactory model fitting. Thus, physical activity was excluded from the model as well. Finally, 5 CR-enhancing factors (i.e., education, early-, mid-, and late-life cognitive activities, and late-life social activity) were used to generate the final CR indicator (eFigure 1, doi.org/10.5061/dryad.59zw3r273).

The loadings of each factor were 0.505 (standard error [SE] 0.021, *p* < 0.001) for education, 0.752 (SE 0.015, *p* < 0.001) for early-life cognitive activity, 0.839 (SE 0.014, *p* < 0.001) for mid-life cognitive activity, 0.592 (SE 0.019, *p* < 0.001) for late-life cognitive activity, and 0.360 (SE 0.024, *p* < 0.001) for late-life social activity. The weight of the corresponding coefficient of the 5 factors from the SEM was 0.060 for education, 0.812 for early-life, 1.277 for mid-life, 0.383 for late-life cognitive activities, and 0.211 for late-life social activity. The final predicted value (as score) of the CR for each participant was generated by summing the products of standardized factors scores and the corresponding SEM-factor weights. The CR score ranged from −8.002 to 5.738, with a higher score indicating a greater level of CR. CR indicator (score) was used as both a continuous and categorical variable (tertile: the lowest [as reference], middle, and the highest) in the following data analyses.

### Assessment of Cognitive Domains, Mild Cognitive Impairment, and Dementia

Cognitive function was assessed via a battery of 21 cognitive performance tests administered at baseline and each year follow-up, as described previously.^37^ Of the tests, the Mini-Mental State Examination (MMSE) was used to describe the cohort and another test (“complex ideas”) was only used to classify the diagnosis of cognitive function instead of being used in the analyses. The other 19 tests were used to assess performance in 5 cognitive domains: episodic memory (Word List Memory, Word List Recall, Word List Recognition, immediate and delayed recall of Story A from the Logical Memory subtest of the Wechsler Memory Scale–Revised, immediate and delayed recall of the East Boston Story), working memory (Digit Span Forward and Backward of the Wechsler Memory Scale–Revised and Digit Ordering), semantic memory (Verbal Fluency, Boston Naming Test, the National Adult Reading Test), perceptual speed (oral version of the Symbol Digit Modalities Test, Number Comparison, 2 indices from a modified Stroop Neuropsychological Screening Test), and visuospatial ability (Judgment of Line Orientation, Standard Progressive Matrices). All the raw scores of the tests were individually converted to *z* scores, which were further averaged to yield a global cognitive function,^38,39^ with higher scores indicating better cognitive function.

The assessments of dementia and mild cognitive impairment (MCI) were based on a 3-stage procedure involving computer scoring of cognitive tests, clinical judgment by a neuropsychologist, and diagnostic classification by an experienced clinician.^40,41^ Clinical diagnosis of dementia was based on the criteria of the joint working group of the National Institute of Neurologic and Communicative Disorders and Stroke and the Alzheimer's Disease and Related Disorders Association.^40^ The diagnosis of MCI was rendered for persons who were judged to have cognitive impairment but not meet the criteria for dementia by the neuropsychologist and clinician.^41^

### Assessment of Brain Pathologies

Brain pathologies were evaluated in 648 deceased participants (median postmortem interval 7 hours [interquartile range (IQR) 5.75–9.25]). The neurologists followed a standard protocol for tissue preservation, tissue sectioning, and quantification of pathologic findings, as detailed in a previous study.^42^ Global Alzheimer disease (AD) pathology burden (including diffuse and neuritic plaques and neurofibrillary tangles) was quantified^42^ and dichotomized as low or high based on its median. Chronic infarcts (including gross infarcts and microinfarcts),^43^ cerebral vascular disease pathology (including atherosclerosis, arteriolosclerosis, and cerebral amyloid angiopathy), Lewy bodies, and typical hippocampal sclerosis^5^ were assessed as present or absent.

### Statistical Analysis

The characteristics of study participants at study entry by different CR groups were compared using one-way analysis of variance/Wilcoxon rank-sum tests for continuous variables and χ^2^ tests for categorical variables.

The β coefficients and 95% confidence intervals (CIs) of the associations between CR indicator (including continuous and categorical CR) and annual change in global cognitive function and 5 cognitive domains were estimated using linear mixed-effect models, with follow-up time (in years) as the time scale. The fixed effect included CR indicator, follow-up time, and their interaction. To allow for the individual differences at baseline and over time, we included random effects for the intercept and slope for the time in the model. When CR was used as a continuous variable, each point change of cognitive function responded to a unit change of CR indicator. When CR indicator was used as a categorical variable (tertile), the points change in cognitive function were corresponding to the middle or highest CR in comparison to the lowest. Age, sex, BMI, smoking status, alcohol consumption, physical activity, hypertension, diabetes, heart disease, stroke, and *APOE* ε4 were considered as potential confounders. To further explore the role of *APOE* ε4 in the relationship between CR indicator and cognitive function, an interaction term between CR and *APOE* ε4 status was included in the models first; then, stratified analysis was performed according to *APOE* ε4.

Among the participants with autopsies, the odds ratios (ORs) with 95% CI of the relationships between CR indicator and brain pathologies were estimated using multinomial logistic regression. Linear mixed-effect models were used again to explore the association of CR indicator with cognitive function by different levels of brain pathologies. In the sensitivity analysis, we repeated the linear mixed-effect models by excluding 433 individuals with MCI at baseline and imputing missing values for covariates. A 2-tailed *p* value < 0.05 was considered to be statistically significant for all tests. All statistical analyses were performed using Stata SE 15.0 for Windows (StataCorp).

### Data Availability

Data requests can be made at radc.rush.edu or available from the corresponding author on reasonable request. The results of supplementary analyses are available from Dryad (eTables 1–13 and eFigure 1, doi.org/10.5061/dryad.59zw3r273).

## Results

### Characteristics of the Study Population at Baseline

Among the 1,697 dementia-free participants (mean age: 79.6 ± 7.5 years, female: 75.7%), 560 (33%), 560 (33%), and 577 (34%) were included in the lowest, middle, and highest CR groups, respectively. Compared to the lowest CR group, participants with the highest CR were more likely to have physical activity, better global cognitive function, and higher scores of MMSE, episodic memory, semantic memory, working memory, visuospatial ability, and perceptual speed, but less likely to have diabetes. There were no significant differences in terms of age, sex, BMI, *APOE* ε4, hypertension, heart disease, or stroke among the 3 groups (Table 1).

**Table 1:**
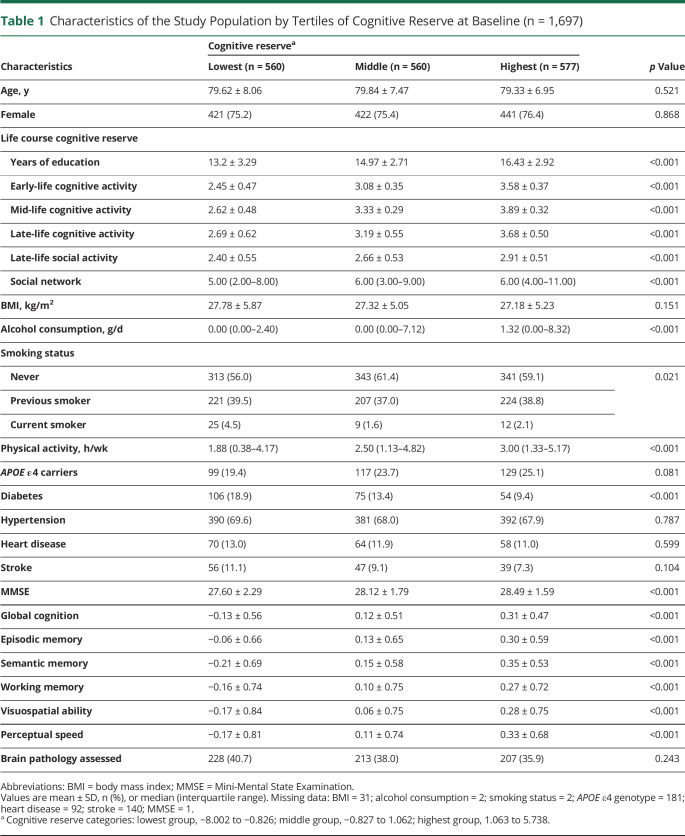
Characteristics of the Study Population by Tertiles of Cognitive Reserve at Baseline (n = 1,697)

### Relationship Between CR Indicator and Cognitive Decline

At baseline, when the CR indicator was operationalized as a continuous variable, a higher score was associated with better global cognitive function and 5 cognitive domains (episodic memory, semantic memory, working memory, visuospatial ability, and perceptual speed). When the CR indicator was analyzed as a categorical variable (tertiles), participants with the highest CR had better cognitive function in all domains compared to those with the lowest CR.

During the follow-up (median 5.31 years, IQR 2.61–8.80 years), the CR indicator was associated with a slower rate of decline in global cognition, episodic memory, semantic memory, working memory, and perceptual speed over time in multi-adjusted mixed-effect models. Compared to the lowest CR, the highest was associated with a slower decline in global cognition, episodic memory, and working memory over the follow-up. However, no statistically significant association of the middle CR with cognitive function decline was shown (Table 2).

**Table 2:**
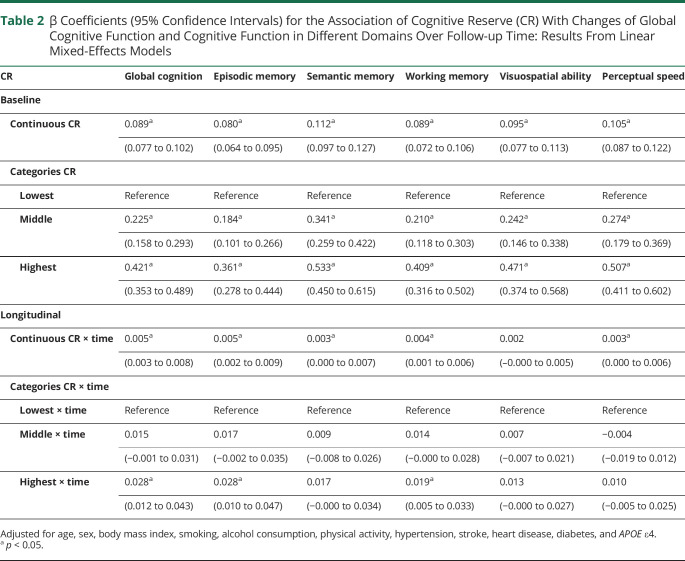
β Coefficients (95% Confidence Intervals) for the Association of Cognitive Reserve (CR) With Changes of Global Cognitive Function and Cognitive Function in Different Domains Over Follow-up Time: Results From Linear Mixed-Effects Models

There was no statistically significant interaction between CR indicator and *APOE* ε4 on cognitive decline (all *p* values > 0.05). However, in the stratified analysis by *APOE* ε4, the associations of higher CR with a slower decline in global and domain-specific cognitive function were present only among *APOE* ε4 noncarriers (Table 3).

**Table 3:**
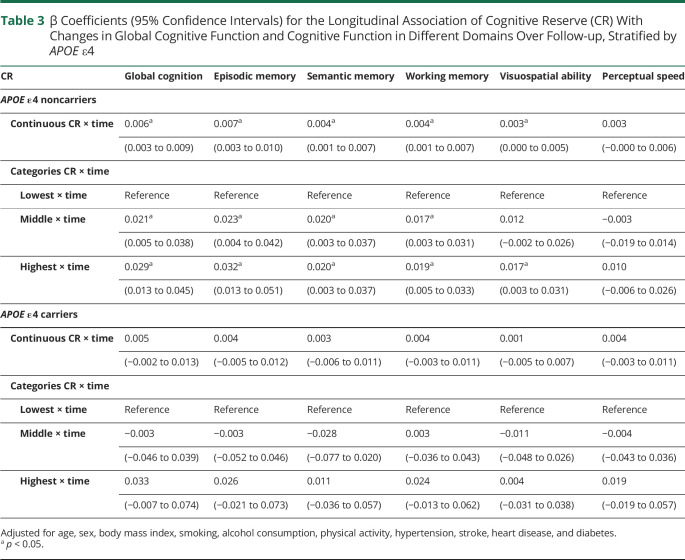
β Coefficients (95% Confidence Intervals) for the Longitudinal Association of Cognitive Reserve (CR) With Changes in Global Cognitive Function and Cognitive Function in Different Domains Over Follow-up, Stratified by *APOE* ε4

### Role of Brain Pathologies in the CR–Cognitive Decline Association

During the follow-up, 795 participants died; of them, 648 (81.5%) underwent autopsies. In multi-adjusted multinomial logistic regression analyses, the highest CR was related to a lower burden of global AD pathology (OR 0.66, 95% CI 0.45–0.98), neuritic plaque (OR 0.75, 95% CI 0.57–1.00, *p* = 0.047), and gross infarcts (OR 0.47, 95% CI 0.29–0.76), but not related to other brain pathologies compared to the lowest CR (Table 4). When the CR indicator was used as a continuous variable, similar results were shown (eTable 2, doi.org/10.5061/dryad.59zw3r273).

**Table 4:**
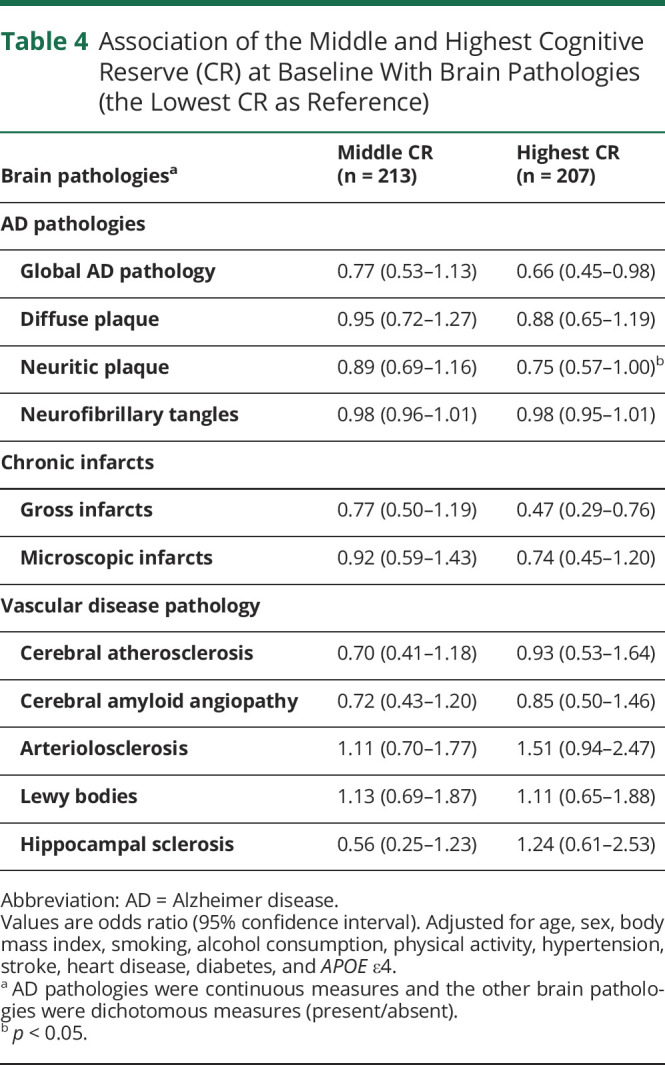
Association of the Middle and Highest Cognitive Reserve (CR) at Baseline With Brain Pathologies (the Lowest CR as Reference)

**Figure 2:**
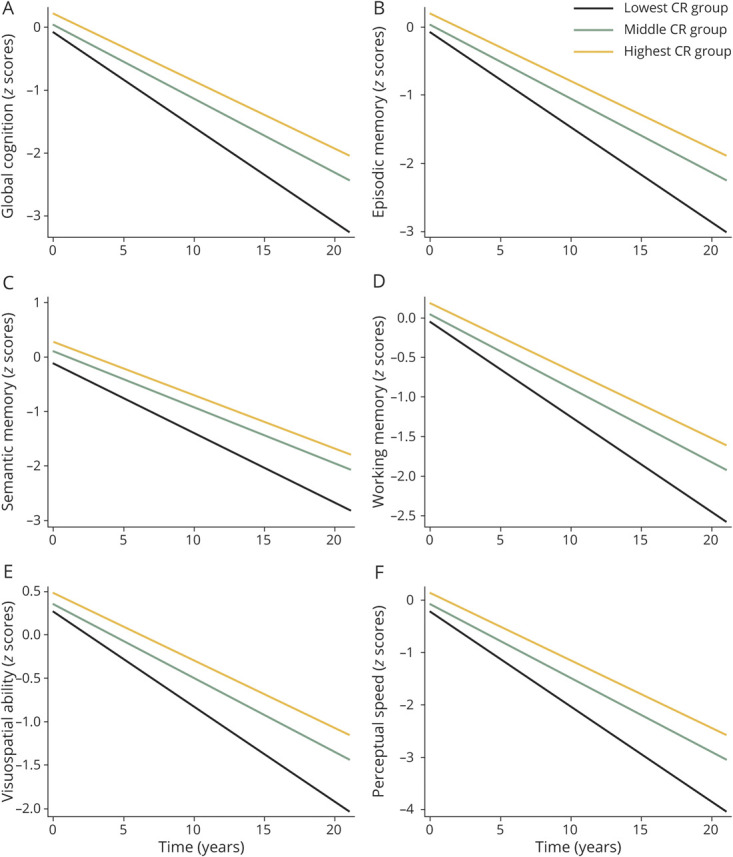
Cognitive Trajectories in Global Cognition and Different Domains by Cognitive Reserve (CR) in Tertiles Further Adjusted by Brain Pathology (A–F) Trajectories represent β coefficients from linear mixed-effect models adjusted for age, sex, body mass index, smoking, alcohol consumption, physical activity, hypertension, stroke, heart disease, diabetes, *APOE* ε4*,* global Alzheimer disease (AD) pathology, and gross infarcts (the lowest CR as reference).

Among the participants who underwent autopsies, the association between the highest CR and slower decline in global cognitive function, episodic memory, working memory, or visuospatial ability remained significant after additional adjustment for brain pathologies (Figure 2 and eTables 3 and 4, doi.org/10.5061/dryad.59zw3r273).

**Figure 3:**
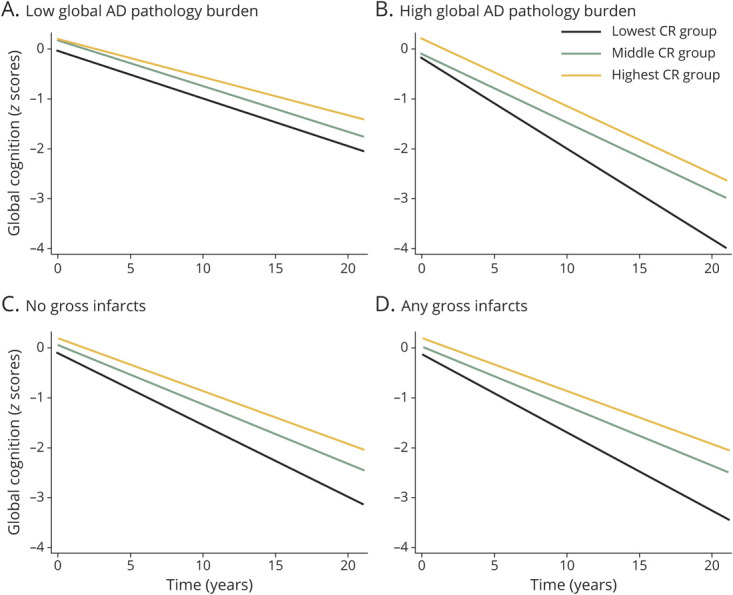
Cognitive Trajectories in Global Cognitive Function by Cognitive Reserve (CR) (Lowest CR as Reference) and Brain Pathology (A–D) Trajectories represent the values of β coefficients from linear mixed-effect models adjusted for age, sex, body mass index, smoking, alcohol consumption, physical activity, hypertension, stroke, heart disease, diabetes, and *APOE* ε4. AD = Alzheimer disease.

In stratified analysis by the level of brain pathologies, the effect of the highest CR indicator on a slower decline in global cognitive function, but not domain-specific cognitive function, remained significant in participants with high AD pathology or gross infarcts (Figure 3 and eTables 5–6, doi.org/10.5061/dryad.59zw3r273).

### Supplementary Analysis

Similar results to those from the main analyses were obtained when we repeated the analysis by (1) excluding 433 individuals with MCI at baseline (eTable 7, doi.org/10.5061/dryad.59zw3r273), (2) performing multiple imputations for missing values of covariates (n = 434) (eTable 8, doi.org/10.5061/dryad.59zw3r273), (3) removing late-life measures (late-life cognitive activity and social activity), mid-life measure (mid-life cognitive activity), or early-life measures (education and early-life cognitive activity) from SEM, separately (eTables 9–11, doi.org/10.5061/dryad.59zw3r273), (4) using individual CR-related factors (eTable 12, doi.org/10.5061/dryad.59zw3r273), and (5) in addition adjusting for survival status during the follow-up (eTable 13, doi.org/10.5061/dryad.59zw3r273).

## Discussion

In the community-based longitudinal study among dementia-free older adults with neuropathologic data available in a subsample, we found that (1) high CR indicator composed of education, early-life, mid-life, and late-life cognitive activities and late-life social activity predicted preserved cognitive functioning in global cognition, episodic memory, and working memory; and (2) the association of high CR indicator with a slower decline in cognitive function remained significant in the presence of high AD pathology or gross infarcts.

In the past 10 years, since the CR concept emerged, the research to explore the relationship between CR and cognitive decline has been stimulated with widespread interest. Thus far, the majority of cross-sectional studies on the association between individual CR-related factors and cognitive function have consistently documented that people with higher education attainment, frequent participation in cognitively stimulating activities, or social activities may have better cognitive function.^7-9,16,17,44^ However, results from longitudinal studies addressing the association of CR-related factors with the decline in cognitive function are inconsistent. In a 12-year follow-up study, educational years were not related to rates of change over time of any cognitive domain.^14^ Another study of older Americans (≥70 years) has shown that a higher level of education is linked to a faster cognitive decline of verbal memory.^12^ Besides, some studies have demonstrated that more frequent cognitive activity is associated with reduced rates of decline in episodic memory, semantic memory, and perceptual speed, but not working memory or visuospatial ability,^18^ and that more frequent cognitive activity in early life^21^ or across the lifespan^42^ is associated with slower late-life cognitive decline. However, cognitive activity is not related to change in cognitive performance over 2–3 years follow-up in another report.^19^ Furthermore, the longitudinal study within the Betula project has shown that social activity is a predictor of episodic memory, but there is no influence of social activity on semantic memory.^20^ Possible explanations for the discrepancies could be the differences in the assessments of CR indicators, follow-up time, and characteristics of study populations among these studies.

Heretofore, the evidence on the relationship between the composite index of CR and cognitive decline has been relatively limited. In our study, we used SEM to yield the CR indicator based on educational and mentally stimulating activities throughout the life course.^31^ To our knowledge, this is the first study to explore the association between life course CR indicator and decline of cognitive function in specific domains. We found that a higher CR indicator was associated with not only better baseline cognitive function, but also preserved cognitive functioning in global cognition, episodic memory, and working memory longitudinally.

It is well-known that *APOE* ε4 is an established risk factor for cognitive decline and the development of dementia.^45^ In the current study, we found that the association of higher CR indicator with slower cognitive decline was present only among *APOE* ε4 noncarriers, but not in *APOE* ε4 carriers. However, the interaction between CR indicator and *APOE* ε4 on cognitive decline was not significant. These results might be due to the fact that *APOE* ε4 carriers who had developed dementia were excluded at baseline, or insufficient power caused by the small sample of *APOE* ε4 carriers in our study.

The relationship between brain pathology and cognitive function is increasingly recognized to be complex. The role of brain pathology in the CR–dementia association has been explored, and 2 hypotheses have been proposed. One is that CR might be directly associated with neuropathology, such as reducing the deposition of β-amyloid in aging.^26^ Another suggests that CR may delay the onset of dementia by compensating for or coping with brain pathology through other pathways,^27,46^ such as a higher neuronal density and greater cortical thickness,^47^ thus presenting the ability to effectively use the brain network.^3^ To explore the role of brain pathologies in the CR–cognitive decline association, in the second part of our study, we found that the highest CR was weakly related to global AD pathology and gross infarcts, suggesting that CR might exert a slight neuroprotective effect in cognitively normal older adults. However, the association of the highest CR indicator with preserved cognitive function over time was still significant after adjusting for these brain pathologies, and this association remained significant even among participants with high global AD pathology or gross infarcts. Our results may support the 2 hypotheses mentioned above.

The possible mechanisms underlying the association between CR and cognitive function are not clear. Environmental enrichment has been related to a variety of neuroplastic responses (such as the formation of new neurons and synapses) in brain regions that are critically involved in cognitive functioning in animals.^48^ In humans, education promotes cognitive processing and brain networking, leading to slower cognitive declines in the presence of neuropathology.^49^ Frequent mental activity and social engagement may contribute to structural and functional reorganization, making neural systems involved in the activities less disrupted by AD pathology.^50^ Mental activity and social engagement may also increase synaptic density in the cortex through stimulation, so that cognitive functioning in unaffected neurons might be able to compensate for the loss of function in affected brain areas in a pathologic process.^20^ Thus, individuals with high CR may develop more alternative neural networks to maintain cognitive function. However, further research is needed to identify the structural, biochemical, and molecular mechanisms of the brain's ability to withstand brain pathologies.

A notable strength of our study is the community-based prospective cohort with a relatively large sample size and long-term follow-up. Furthermore, the use of latent variable could capture CR-related factors from different periods over the life course. Nevertheless, some limitations of our study should be pointed out. First, the participants were volunteers with a mean age of 79.6 years at study entry. Thus, caution is required when generalizing our findings, especially the results regarding brain pathology, to the younger population. Second, the CR-related factors were ascertained through retrospective self-reported information, which might lead to some measurement errors. However, using the SEM to extract the CR indicator might attenuate some of the errors. Furthermore, information on occupation was not available, and thus could not be considered in CR indicator. Third, the brains of the participants were obtained and autopsied when the participants were deceased, and the exact time brain pathologies occurred was unknown. Finally, all participants were living in urban areas, thus caution is needed when generalizing our findings to older adults living in rural areas. In addition, data on nutrition were not available for all participants, and nutrition could not be taken into account in the data analysis.

This study provides evidence that high CR indicator assessed by education; early-life, mid-life, and late-life cognitive activities; and late-life social activity may preserve cognitive functioning over time. We further found that the association of high CR with a slower cognitive decline remained significant in the presence of high AD pathology or gross infarcts. Our findings underscore the importance of educational and mentally stimulating activities throughout the lifespan for preserved cognitive function in late life. Further large population-based longitudinal studies are required to verify the effect of CR on cognitive decline and brain pathologies.

## Glossary

AD: Alzheimer disease
BMI: body mass index
CI: confidence interval
CR: cognitive reserve
IQR: interquartile range
MCI: mild cognitive impairment
MMSE: Mini-Mental State Examination
OR: odds ratio
SE: standard error
SEM: structural equation modeling
